# Autophagy Activation by *Crepidiastrum Denticulatum* Extract Attenuates Environmental Pollutant-Induced Damage in Dermal Fibroblasts

**DOI:** 10.3390/ijms20030517

**Published:** 2019-01-26

**Authors:** Seok Jeong Yoon, Chae Jin Lim, Hwa-Jee Chung, Joo-Hwan Kim, Yang Hoon Huh, Keedon Park, Sekyoo Jeong

**Affiliations:** 1 R&D Center, Incospharm Corp, 328 Techno-2-ro, Yuseong-gu, Daejeon 34036, Korea; sjyoon@incospharm.com (S.J.Y.); cjlim@incospharm.com (C.J.L.); hjchung@incospharm.com (H.-J.C.); kdpark@incospharm.com (K.P.); 2 Department of Life Science, Gachon University, 1342 Seongnamdaero, Sujeong-gu, Seongnam, Gyeonggi-do 13120, Korea; kimjh2009@gachon.ac.kr; 3 Electron Microscopy Research Center, Korea Basic Science Institute, 162 Yeongudanji-ro, Ochang-eup, Cheongwon-gu, Cheongju, Chungbuk 28119, Korea; hyh1127@kbsi.re.kr; 4 School of Cosmetics, Seowon University, 377-3 Musimseo-ro, Seowon-gu, Cheongju, Chungbuk 28674, Korea

**Keywords:** autophagy, anti-inflammation, anti-pollution, *Crepidiastrum denticulatum* extract, skin barrier

## Abstract

Pollution-induced skin damage results in oxidative stress; cellular toxicity; inflammation; and, ultimately, premature skin aging. Previous studies suggest that the activation of autophagy can protect oxidation-induced cellular damage and aging-like changes in skin. In order to develop new anti-pollution ingredients, this study screened various kinds of natural extracts to measure their autophagy activation efficacy in cultured dermal fibroblast. The stimulation of autophagy flux by the selected extracts was further confirmed both by the expression of proteins associated with the autophagy signals and by electron microscope. *Crepidiastrum denticulatum* (CD) extract treated cells showed the highest autophagic vacuole formation in the non-cytotoxic range. The phosphorylation of adenosine monophosphate kinase (AMPK), but not the inhibition of mammalian target of rapamycin (mTOR), was observed by CD-extract treatment. Its anti-pollution effects were further evaluated with model compounds, benzo[a]pyrene (BaP) and cadmium chloride (CdCl_2_), and a CD extract treatment resulted in both the protection of cytotoxicity and a reduction of proinflammatory cytokines. These results suggest that the autophagy activators can be a new protection regimen for anti-pollution. Therefore, CD extract can be used for anti-inflammatory and anti-pollution cosmetic ingredients.

## 1. Introduction

In addition to the well-known intrinsic and extrinsic factors inducing skin aging, such as chronological changes and solar irradiation, environmental factors, including ambient particulate matter (PM) or infrared irradiation, recently emerged as important deleterious factors for skin aging. PM is known to be consisted of mainly organic chemicals and inorganic constituents, including polycyclic aromatic hydrocarbons (PAHs) and heavy metals, respectively. It was reported that cutaneous exposure to PAHs and heavy metals results in epidermal cytotoxicity [1,2,3], harmful impacts on dermal extracellular matrix proteins [4], inflammatory responses [5], and impairment on skin barrier functions [6]. PM is increasing in the ambient air from industrialization and urbanization trends, and their detrimental effects on skin health has made them one of the most serious environmental pollution problems for skin disease [7], which is supported by the aggravation of eczema and itching symptoms in atopic dermatitis from PM exposure [8]. Other studies also reported that chronic exposure to traffic-related PM is associated with premature skin aging, shown as increased pigment spots and more distinct wrinkles in urban areas [9].

One of the most common detrimental mechanisms of PM and other extrinsic skin aging factors is excessive oxidative stress on skin, forming oxidized biomolecules or organelles. These oxidized molecules induce cytotoxic damage, cellular dysfunctions, or structural impairments. Therefore, enhancing the cellular anti-oxidant potency, either using anti-oxidant molecules [10] or upregulating cellular anti-oxidant enzymes’ expressions [11], is one of the most widely used skin anti-aging strategies. While various kinds of small molecules, natural extracts, or proteins have been developed as anti-aging ingredients, most of the ingredients showed their efficacy based on direct anti-oxidant effects (i.e., the removal of reactive oxygen species (ROS) through direct chemical interaction). Recently, autophagy signaling in skin, and its physiological roles in skin homeostasis, have been extensively investigated. Along with the crucial roles in epidermal differentiation process [12], potential involvement in skin barrier functions, inflammation [13], and even in the aging process [14] have been suggested for the autophagy process. Recently, we also reported that the topical application of the autophagy activating molecule significantly reduced the oxidative stress marker molecule, carbonylated proteins, in stratum corneum clinically [15]. Considering the crucial roles of autophagy in the cellular anti-oxidant system [16,17], it can be postulated that autophagy activating molecules can also prevent PM-induced damage to the skin. In this study, we tried to identify a novel natural extract with an autophagy activating efficacy in cultured dermal fibroblast, and evaluated its potential benefits as an anti-aging ingredient. A cytoprotective effect against PM and an anti-inflammatory activity were also investigated in a cultured epidermal keratinocyte.

## 2. Results

**Figure 1:**
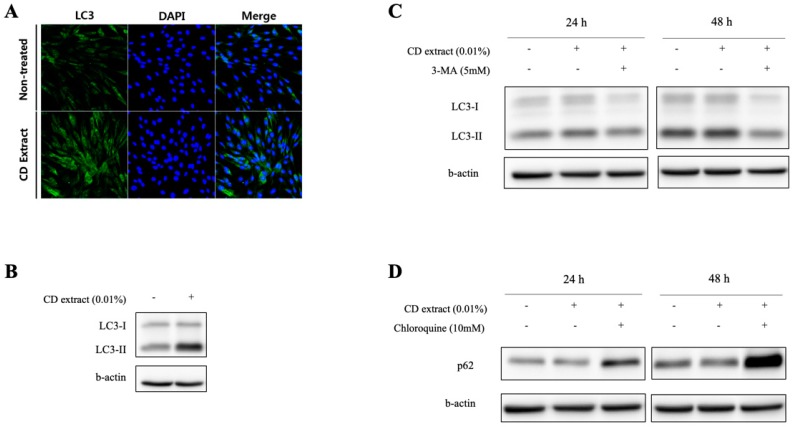
Autophagy induction by *Crepidiastrum denticulatum* (CD) extract in dermal fibroblast. Treatment of CD extract induced increased expression of LC3 positive puncta structures in cultured human dermal fibroblast cells (blue: 4′,6-Diamidino-2-phenylindole (DAPI)) (**A**) (magnification X200). Representative blots from triplicate showed that the transition of the LC3-II protein was upregulated by CD extract treatment in cultured human dermal fibroblast cells (**B**), which was blocked by the co-treatment of autophagy inhibitor of 3-3-Methyladenine (3-MA) (**C**). Reduction of the p62 protein, as a marker for autophagy response, was also blocked by chloroquine (CQ) treatment (**D**). + means treated and – means non-treated.

The preliminary screening of various natural extracts for identifying the autophagy stimulating compounds resulted in a few candidates, and, in this study, the ethanolic extract of *Crepidiastrum denticulatum* (CD) was selected for the further investigation, based on its high activity and low cytotoxicity. The preliminary cytotoxicity measurements with 3-(4,5-Dimethylthiazol-2-yl)-2,5-Diphenyltetrazolium Bromide (MTT) assay showed that 0.01% (*w*/*v*) of the CD extract showed no cytotoxicity in both the dermal fibroblast and epidermal keratinocyte, and further experiments were performed based on this concentration. Firstly, the autophagy stimulation of CD extract was examined by immunofluorescence staining. As shown in Figure 1A, an increased expression of Microtubule-associated protein light chain 3 (LC3) proteins was observed in cultured human dermal fibroblast (HDF) cells after CD extract treatment. Punta structures at the perinuclear area further suggested an increased conversion of LC3-II proteins, which was confirmed by the protein quantitation result (Figure 1B). The co-treatment of either an early stage autophagy inhibitor, 3-methyladenine (3-MA), or a late stage autophagy inhibitor, chloroquine (CQ), resulted in a decreased conversion into a LC3-II protein (Figure 1C), or an increased amount of p62 protein (Figure 1D), respectively. This suggested that intracellular changes by CD extract treatment are, at least in part, through the modulation of autophagy activity.

**Figure 2:**
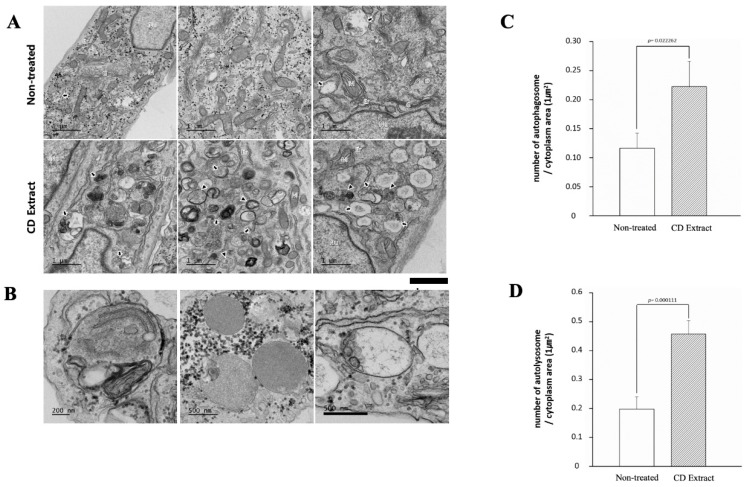
Transmission electron microscopic images of dermal fibroblast treated without or with CD extract. Increased formation of autophagosome and autolysosome by CD extract treatment in cultured human dermal fibroblasts was observed by transmission electron microscope (TEM) (**A**). Early autophagosome (left panel), autophagosome (center panel), and autolysosome (right panel) were observed in the CD extract treated cells (**B**). A significantly increased number of autophagosomes (**C**) and autolysosomes (**D**) in the unit cytoplasmic area further confirmed the activation of autophagy response by CD extract. Arrowhead: autophagosome; arrow: autolysosome; Nu: nucleus; ER: endoplasmic reticulum; G: Golgi apparatus; M: mitochondria.

The stimulation of autophagy responses by CD extract in the dermal fibroblast was further investigated morphologically, using a transmission electron microscope (TEM). The TEM observation showed an increased number of both autophagosomes and autolysosomes in the CD extract treated cells (Figure 2A,B). Compared with the non-treated cells, the number of autophagosomes per unit cytoplasmic area significantly increased from 0.12 to 0.23 (Figure 2C), and the number of autolysosomes also significantly increased from 0.2 to 0.43 (Figure 2D).

**Figure 3:**
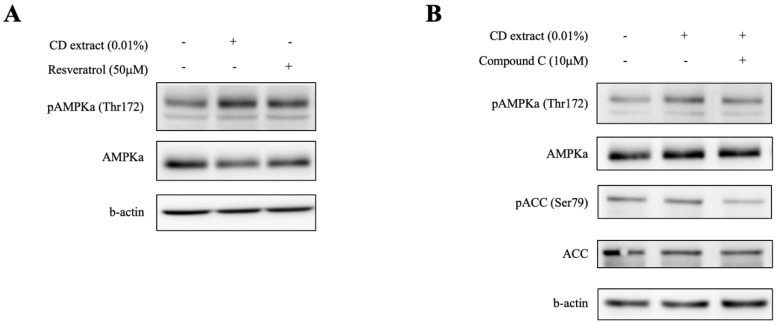
Activation of the adenosine monophosphate kinase (AMPK) signaling pathway by CD extract in dermal fibroblast. Representative blots from triplicate showed that the treatment of either CD extract or resveratrol resulted in an increased phosphorylation of AMPKa in the cultured human dermal fibroblast cells (**A**). The co-treatment of Compound C, as an AMPK inhibitor, blocked the phosphorylation of AMPKa and acetyl-CoA carboxylase (ACC) by CD extract (**B**). + means treated and – means non-treated.

In order to explore the mechanism of autophagy activation by CD extract, the changes of autophagy-related signaling proteins, that is, mTOR (mammalian target of rapamycin) and adenosine monophosphate (AMP) kinase, were investigated. An increased phosphorylation of AMPK was observed in the CD extract treated dermal fibroblast, of which the level was similar to that of the resveratrol treatment (Figure 3A), but there was no noticeable change in mTOR phosphorylation. The co-treatment of Compound C, an AMPK inhibitor, blocked the phosphorylation of AMPK and acetyl-CoA carboxylase (ACC), which suggests that the phosphorylation of AMPK, at least in part, underlies the autophagy stimulating mechanism of the CD extract.

**Figure 4:**
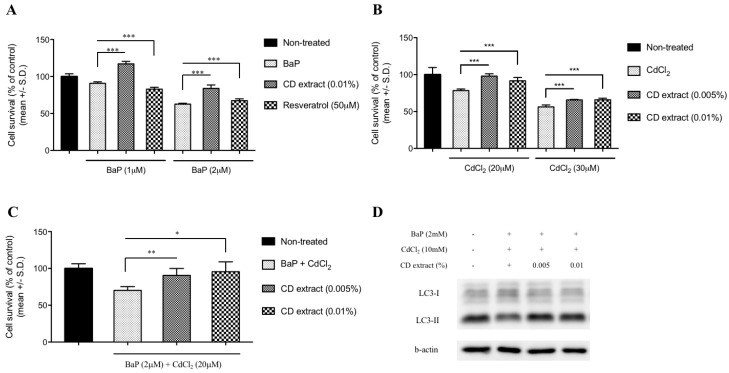
Cytoprotective effects of CD extract in dermal fibroblast. Benzo[a]pyrene (**A**) and cadmium chloride (**B**) treatment induced a dose-dependent cytotoxicity on cultured human dermal fibroblast, respectively, which was significantly alleviated by the co-treatment of the CD extract (**A**,**B**) and resveratrol (**A**). Cytotoxicity induced by simultaneous treatment of benzo[a]pyrene and cadmium chloride were further blocked by CD extract co-treatment (**C**). Representative blots from triplicate showed that decreased expression of LC3-II protein by benzo[a]pyrene and cadmium chloride treatment were overcome by the co-treatment of the CD extract (**D**). + means treated and – means non-treated. *: *p* < 0.05, **: *p* < 0.01, ***: *p* < 0.001

Based on previous reports suggesting the protective effects of autophagy activators on cytotoxic stresses, the cytoprotective effects of the CD extract were further examined in a cultured dermal fibroblast. In order to simulate the environmental stresses in urban sites, benzo[a]pyrene (BaP) and cadmium chloride were used as organic and heavy metal pollutants, respectively. While a dose-dependent cytotoxicity was observed in the BaP treated human dermal fibroblast cells, the co-treatment of CD extract significantly prevented cell death. However, the treatment of resveratrol did not show preventive effects against BaP (Figure 4A). The treatment of cadmium chloride also induced dose-dependent cytotoxic changes, and the co-treatment of the CD extract significantly reduced the cytotoxicity (Figure 4B). The simultaneous treatment of BaP and cadmium chloride also resulted in a significant cytotoxicity, which was alleviated by CD extract treatment in a dose-dependent manner. In order to assess whether the autophagy process is involved in the cytoprotective activity of CD extract, the expression of LC3 proteins were analyzed. While the BaP/cadmium chloride treatment significantly downregulated the expression of the LC3-II expression, the CD extract treatment reversed the LC3-II expressions (Figure 4D).

**Figure 5:**
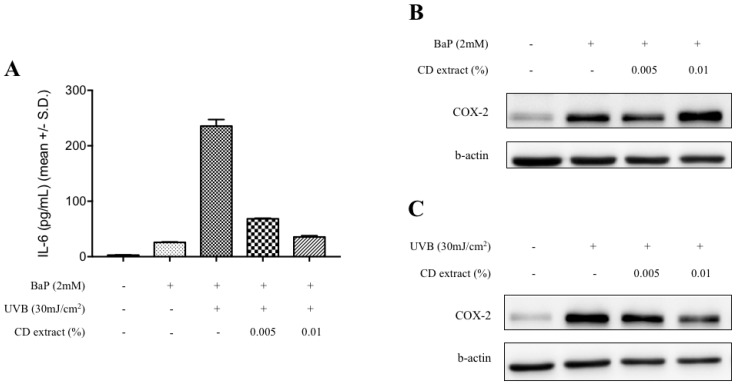
Anti-inflammatory activity of CD extract in epidermal keratinocyte. Dose-dependent reduction of secreted interleukin-6 (IL-6) was observed in the CD-extract treated human epidermal keratinocyte cells (**A**). The benzo[a]pyrene (BaP)-only treated cells showed a slight increase in IL-6 expression, which were not affected by the CD extract treatment. While the CD extract treatment did not result in a significant blockade of cyclooxygenase-2 expression in the benzo[a]pyrene treated human epidermal keratinocyte cells (**B**), the representative blots from triplicate showed that the UVB exposure induced cyclooxygenase-2 expression was noticeably down-regulated by the CD extract treatment in a dose-dependent manner (**C**). + means treated and − means non-treated.

The expression of pro-inflammatory cytokine, interleukin-6 (IL-6), in epidermal keratinocyte was measured, in order to verify whether this cytoprotective activity of the CD extract can provide anti-inflammatory effects. Similar to dermal fibroblast, the CD extract treatment induced the stimulation of autophagic flux in cultured epidermal keratinocyte. In cultured epidermal keratinocytes, the treatment of BaP significantly stimulated the IL-6 expression in the culture media, and its level was further increased by simultaneous treatment with UVB irradiation (Figure 5A). As expected, the co-treatment of the CD extract down-regulated the IL-6 expression in a dose-dependent manner, and the expression of cyclooxygenase-2 enzyme showed the same changes (Figure 5B,C), which suggests an anti-inflammatory activity of the CD extract against BaP and UVB irradiation.

## 3. Discussion

As a consequence of industrialization and urbanization, increased exposure to solar irradiation and a worsening of ambient air pollution are suggested as one of the most notorious stresses for skin health [9,18]. Skin, as the outermost organ, relentlessly faces the external stresses and mediates the homeostatic responses against environmental changes. As a first line of defense against external stresses, the skin has developed diverse barrier functions, including an epidermal permeability barrier, antimicrobial barrier, and anti-oxidant barrier [19]. Several kinds of signaling mechanisms, including calcium ion concentration, retinoic acid receptor (RAR), and vitamin-D receptor signaling, are reported as regulating the differentiation of keratinocytes, and autophagy is also involved in the epidermal differentiation process [12]. It has also been reported that autophagy plays important roles in inflammation, the anti-oxidant system [20], and skin barrier formation [13].

The assessment of autophagy signaling in cells requires measuring the expression of autophagy marker proteins, such as LC3-II, as well as observing the autophagic flux changes using electron microscopy or appropriate inhibitors [21]. In this study, we observed the expression of autophagic vacuoles using immunohistochemical staining in cultured dermal fibroblast as a screening step. From the screening experiments, the ethanolic extract of *Crepidiastrum denticulatum* (CD) showed the most distinct puncta structures near the peri-nuclear area, which represents the initiation of the autophagic vacuole formation. Changes in the autophagy marker proteins, LC3-II and p62, also supported that the CD extract stimulates the autophagy process in a cultured human dermal fibroblast. The simultaneous treatment of autophagy inhibitors with a different point of action, using 3-Methyladenine (3-MA) as an inhibitor of type III Phosphatidylinositol 3-kinases (PI-3K), or chloroquine as an inhibitor of lysosomal degradation, significantly blocked the expression changes, respectively, which further supports that the CD extract activates the autophagy process in the dermal fibroblast. Ultrastructural observation using transmission electron microscope also revealed a more than two-fold increase in both autophagosomes (0.12 to 0.23) and autophagolysosomes (0.2 to 0.43) in the CD extract treated cells. The canonical members of the autophagy signaling pathway, including ULK1/2 (UNC-51-like kinases 1/2), ATG13, and beclin 1(BECN1), are known to be activated by nutrient deprivation, mainly through the inhibition of the mammalian target of the rapamycin (mTOR) protein, resulting in the translocation of the mTOR substrate complex from the cytosol to the endoplasmic reticulum (ER), or through the activation of a nutrient kinase, such as adenosine monophosphate kinase (AMPK) [22]. While the CD extract treatment did not induce noticeable change in the mTOR protein phosphorylation, an increased phosphorylation of AMPK by was observed in the cultured dermal fibroblast, which was blocked by the co-treatment of Compound C, as an AMPK inhibitor. These results also suggest that the CD extract activates the autophagy signal, at least in part, through AMPK activation, but not through mTOR interaction.

Previous studies provided detoxification and antioxidant activities of *Crepidiastrum denticulatum* (CD) extract showing in vitro chemopreventive effects [23] and in vivo hepatoprotective effects against alcohol-induced liver damage [24]. With its potential benefits as an anti-inflammatory and anti-oxidant ingredient for topical application, however, little has been reported about CD extract’s activity in skin. In this study, we investigated the effects of the CD extract on dermal fibroblast and epidermal keratinocyte, and also explored its working mechanism, focusing on the autophagy process. In order to verify whether the CD extract has a cytoprotective activity against ambient pollution, benzo[a]pyrene (BaP) and cadmium chloride, as a model compound for organic and heavy metal pollutants, respectively, were selected [25,26]. As a result, the co-treatment of CD extract significantly attenuated the BaP and cadmium-induced cytotoxicity. Interestingly, resveratrol, which is a well-known autophagy activator, did not show cytoprotective effects. While there are many reports addressing the beneficial effects of resveratrol as an anti-inflammatory, anti-cancer, and anti-aging ingredient [27], cytotoxicity on a culture keratinocyte was also reported [28], which is consistent with the present study’s results. The potential involvement of autophagy signaling is also implicated by the decreased expression of the LC3-II protein by BaP and cadmium mixture treated cells, which was restored by the co-treatment of the CD extract. To further confirm the anti-inflammatory activity of the CD extract, the expression of IL-6, as a marker cytokine in dermal inflammation [29], was measured. The co-treatment of BaP and UV irradiation strongly upregulated the expression of the IL-6 protein, which was blocked by the CD extract treatment in a dose dependent manner. Measuring the expression of the cyclooxygenase-2 (COX-2) protein also showed similar changes using the BaP treatment. In accordance with previous studies that reported a down-regulation of COX-2 expression by autophagy activators resveratrol [30] or flavonoid treatment [31], the CD extract treatment dose dependently attenuated the COX-2 expression.

In this study, we tried to develop new anti-pollution ingredients, based on the beneficial effects of the topical autophagy activator. Recently, we reported a novel autophagy activating peptide derivative, which binds to the sirtuin 1 protein, thus activating the cellular autophagy process in the skin [32]. From a clinical evaluation, the anti-aging activity of the developed ingredient, in terms of skin elasticity, was confirmed. Based on many studies suggesting the autophagy activating effects of natural products [33,34], various plant extracts were screened for their autophagy activating efficacy. As a result, the CD extract showed the highest autophagy activating efficacy and expected beneficial effects, such as cytoprotective activity and anti-inflammatory, were also observed. These results not only support the crucial roles of autophagy signal in the skin’s anti-pollution system, but also suggest a new way of developing bioactive ingredients based on autophagy signaling.

## 4. Materials and Methods

### 4.1. Reagents and Antibodies

Resveratrol, 3-(4,5-Dimethylthiazol-2-yl)-2,5-Diphenyltetrazolium Bromide (MTT), 3-Methyladenine (3-MA), Chloroquine (CQ), 4′,6-Diamidino-2-phenylindole (DAPI), poly-L-lysine, Benzo[a]pyrene (BaP), Cadmium chloride, and 3,3′,5,5′ Tetrametyl-benzidine (TMB) were purchased from Sigma-Aldrich (St. Loius, MI, USA). The anti-LC3 antibody was from Sigma-Aldrich and the antibodies against AMPK, phospho-AMPK (Thr 172), acetyl-CoA carboxylase (ACC), and phospho-ACC (ser 79) were from Cell Signaling Technology (Danvers, MA, USA). Anti-COX-2 antibody, anti-p62 antibody, and anti-actin antibody were purchased from Abcam (Cambridge, MA, USA), Santa Cruz Biotechnology, Inc. (Dallas, TX, USA), and Young In Frontier Co., Ltd. (Seoul, South Korea), respectively.

### 4.2. Preparation of Crepidiastrum Denticulatum (CD) Extract

The ethanolic extract of the *Crepidiastrum denticulatum* leaf and stem (harvested from Chunma Mt., Namyangju-si, Kyungki-do, South Korea) was provided by Medicinal Plant Resources Bank, Gachon University (Seongnam-si, South Korea). The brief extraction process was as follows: 50 g of leaf and stem part of *C. denticulatum* was mixed with 1 L of 70% ethanol solution, and the mixture was homogenized for one hour two times. After filtering with filter paper, the filtered solution was collected and 1 L of 70% ethanol solution was further added to the filtered solution. The mixed solution was filtered again with filter paper, and concentrated using low temperature concentrator under −110 °C. The concentrated solution was then frozen under −80 °C for two days and lyophilized. The lyophilized sample was aliquoted and stored under −20 °C until the next experiment. For the in vitro assays, the lyophilized sample was dissolved with a 10% DMSO solution.

### 4.3. Cell Culture

The human dermal fibroblast (HDF), normal human epidermal keratinocyte (NHEK), and culture media used for the study were purchased from Thermo Fisher Scientific (Waltham, MA, USA). HDF was cultured with Medium 106 with a low serum growth supplement (LSGS) kit, and NHEK was maintained with EpiLife media with human keratinocyte growth supplement (HKGS) and 1% antibiotics. The cells were cultured under 37 °C, at a 5% CO_2_ condition.

### 4.4. Cell Viability Assay

In order to measure the cytoprotective effects of the CD extract, the HDF cells were seeded on a 96-well plate (6000 cells/well) and were cultured for 24 h. After 24 h of pre-treatment with a CD extract, the cells were exposed either with BaP alone for three days, or a cadmium chloride/BaP mixture for one or two days. The cell viability was measured using MTT assay.

### 4.5. Protein Expression Measurement

The HDF cells or NHEK cells were seeded on either 6 well (1 × 10^5^ cells / well) or 12 well plate (5 × 10^4^ cells/well), and were cultured for 24 h. The CD extract was treated on the cells for an appropriate time and the cell lysates were harvested. The cell lysates were then separated by SDS-PAGE electrophoresis and the proteins were blotted onto a nitrocellulose membrane. After blocking with 0.5% bovine serum albumin solution containing 0.1% Tween-20, the membrane was incubated with primary antibodies overnight at 4 °C. Secondary antibodies conjugated with horse radish peroxidase were incubated for one hour under room temperature, and the protein quantity was measured using ECL detection reagent (Amersham Biosciences Corp., Little Chalfont, UK) using the ChemiDoc system (Alliance Mini HD 9, Uvitec, Cambridge, UK).

### 4.6. Transmission Electron Microscopy

Transmission electron microscopic works were performed in the Korea Basic Science Institute (Ochang, Chungbuk, South Korea). The HDF cells were seeded on a 100-mm culture dish (8 × 10^5^ cells/dish) and were cultured for 24 h. The CD extract was then treated for 24 h and the cells were sequentially fixed with 2.5% glutaraldehyde and 1% osmium tetroxide on ice for 2 h, and washed with PBS. The tissues were then dehydrated in ethanol and propylene oxide series, embedded in an Epon 812 mixture, and polymerized in an oven at 70 °C for 24 h. The sections acquired from the polymerized blocks were collected on grids, counterstained with uranyl acetate and lead citrate, and examined with a Bio-HVEM system (JEM-1400Plus at 120 kV and JEM-1000BEF at 1000 kV, JEOL, JAPAN). For the statistical analysis, TEM images were randomly selected from non-treated cells and CD extract treated cells, and the number of autophagosomes and autolysosomes per unit area of cytoplasm was calculated.

### 4.7. Autophagy Activity Measurement

In order to measure the change of autophagy activity in the cultured HDF cells, the LC3 protein expression was observed with immunofluorescence staining. The cells were seeded on poly-L-lysine coated-slide with 8 wells (ibidi GmbH, Martinsried, Germany) (3 × 10^4^ cells/well), and cultured for 24 h. The CD extract was then treated for 24 h and the cells were fixed with methanol. After incubation with 5% bovine serum albumin containing a PBS buffer solution for one hour at room temperature, rabbit anti-human LC3 antibody was added, and was stored for 24 h at 4 °C. A Fluorescein isothiocyanate (FITC)-labeled secondary antibody was treated for one hour at room temperature, and the cells were observed using a Confocal LASER microscope (LSM800, Carl Zeiss AG, Oberkochen, Germany).

### 4.8. Cytokine Measurement in Epidermal Keratinocytes

The expression of the interleukin-6 protein was performed using human IL-6 ELISA kit (Becton-Dickson, Franklin Lakes, NJ, USA), according to the manufacturer’s suggestion. Briefly, the captured antibody was diluted with a 0.1 M sodium carbonate buffer solution, and coated onto 96 well plate by incubating overnight at 4 °C. After blocking with 10% fetal bovine serum in a PBS buffer solution for one hour at room temperature, the samples were added to each well and were incubated for 2 h at room temperature. After washing with a PBS buffer five times, a detection antibody and streptavidin-HRP reagent mixture was added to each well and was incubated for one hour at room temperature. Tetramethylbenzidine and hydrogen peroxide solutions were added to each well and incubated for 30 min at room temperature in the dark. After stopping adding to the solution, an absorbance at 450 nm was read using an Epoch microplate reader (Biotek, Winoski, VT, USA).

### 4.9. Statistical Analysis

All of the experiments, otherwise stated, were performed in triplicate. The significance of the differences between the groups was evaluated using a one-way ANOVA test, and *p*-value less than 0.05 was considered significant.

## 5. Conclusions

This study suggest that the topical application of autophagy activators can be a new protection regimen against anti-pollution in skin. Hence, as an autophagy activator, CD extract can be used for anti-inflammatory and anti-pollution ingredients for cosmetic application.

## Abbreviations

CD	*Crepidiastrum denticulatum*
